# Structure of the RNA-dependent RNA polymerase from COVID-19 virus

**DOI:** 10.1126/science.abb7498

**Published:** 2020-04-10

**Authors:** Yan Gao, Liming Yan, Yucen Huang, Fengjiang Liu, Yao Zhao, Lin Cao, Tao Wang, Qianqian Sun, Zhenhua Ming, Lianqi Zhang, Ji Ge, Litao Zheng, Ying Zhang, Haofeng Wang, Yan Zhu, Chen Zhu, Tianyu Hu, Tian Hua, Bing Zhang, Xiuna Yang, Jun Li, Haitao Yang, Zhijie Liu, Wenqing Xu, Luke W. Guddat, Quan Wang, Zhiyong Lou, Zihe Rao

**Affiliations:** 1 Laboratory of Structural Biology, School of Life Sciences, and School of Medicine, Tsinghua University, Beijing, China.; 2 Shanghai Institute for Advanced Immunochemical Studies and School of Life Science and Technology, ShanghaiTech University, Shanghai, China.; 3 State Key Laboratory of Medicinal Chemical Biology, Frontiers Science Center for Cell Response, College of Life Sciences, and College of Pharmacy, Nankai University, Tianjin, China.; 4 State Key Laboratory for Conservation and Utilization of Subtropical Agro-Bioresources, College of Life Science and Technology, Guangxi University, Nanning, China.; 5 School of Life Sciences, Tianjin University, Tianjin, China.; 6 School of Chemistry and Molecular Biosciences, The University of Queensland, Brisbane, QLD, Australia.; 7 National Laboratory of Biomacromolecules, CAS Center for Excellence in Biomacromolecules, Institute of Biophysics, CAS, Beijing, China.

## Abstract

A novel coronavirus [severe acute respiratory syndrome–coronavirus 2 (SARS-CoV-2)] outbreak has caused a global coronavirus disease 2019 (COVID-19) pandemic, resulting in tens of thousands of infections and thousands of deaths worldwide. The RNA-dependent RNA polymerase [(RdRp), also named nsp12] is the central component of coronaviral replication and transcription machinery, and it appears to be a primary target for the antiviral drug remdesivir. We report the cryo–electron microscopy structure of COVID-19 virus full-length nsp12 in complex with cofactors nsp7 and nsp8 at 2.9-angstrom resolution. In addition to the conserved architecture of the polymerase core of the viral polymerase family, nsp12 possesses a newly identified β-hairpin domain at its N terminus. A comparative analysis model shows how remdesivir binds to this polymerase. The structure provides a basis for the design of new antiviral therapeutics that target viral RdRp.

## The COVID-19 RNA-synthesizing machine

Many in the scientific community have mobilized to understand the virus that is causing the global coronavirus disease 2019 (COVID-19) pandemic. Gao *et al.* focused on a complex that plays a key role in the replication and transcription cycle of the virus. They used cryo–electron microscopy to determine a 2.9-angstrom-resolution structure of the RNA-dependent RNA polymerase nsp12, which catalyzes the synthesis of viral RNA, in complex with two cofactors, nsp7 and nsp8. nsp12 is a target for nucleotide analog antiviral inhibitors such as remdesivir, and the structure may provide a basis for designing new antiviral therapeutics.

*Science*, this issue p. 779

## Teaser

The structure of the COVID-19 virus polymerase essential for viral replication provides a basis for the design of new antiviral drugs.

Coronavirus disease 2019 (COVID-19) is caused by a novel coronavirus [severe acute respiratory syndrome–coronavirus 2 (SARS-CoV-2)] that emerged in December 2019 (*1*–*3*) and has since become a global pandemic. COVID-19 virus is reported to be a new member of the betacoronavirus genus and is closely related to severe acute respiratory syndrome–coronavirus (SARS-CoV) and several bat coronaviruses (*4*). Compared with SARS-CoV and Middle East respiratory syndrome–coronavirus (MERS-CoV), COVID-19 virus exhibits faster human-to-human transmission, which lead the World Health Organization to declare a worldwide public health emergency (*1*, *2*).

Coronaviruses (CoVs) employ a multisubunit machinery for replication and transcription. A set of nonstructural proteins (nsps) produced as cleavage products of the ORF1a and ORF1ab viral polyproteins (*5*) assembles to facilitate viral replication and transcription. A key component, the RNA-dependent RNA polymerase [(RdRp), also known as nsp12], catalyzes the synthesis of viral RNA and thus plays a central role in the replication and transcription cycle of COVID-19 virus, possibly with the assistance of nsp7 and nsp8 as cofactors (*6*). Therefore, nsp12 is considered a primary target for nucleotide analog antiviral inhibitors such as remdesivir, which shows potential for the treatment of COVID-19 viral infections (*7*, *8*). To inform drug design, we determined the structure of nsp12, in complex with its cofactors nsp7 and nsp8, by cryo–electron microscopy (cryo-EM) using two different protocols: one in the absence of dithiothreitol (DTT) (dataset 1) and the other in the presence of DTT (dataset 2).

The bacterially expressed full-length COVID-19 virus nsp12 (residues S1 to Q932) was incubated with nsp7 (residues S1 to Q83) and nsp8 (residues A1 to Q198), and the complex was then purified (fig. S1). Cryo-EM grids were prepared using this complex, and preliminary screening revealed excellent particle density with good dispersion. After the collection and processing of 7994 micrograph movies, we obtained a 2.9-Å resolution three-dimensional reconstruction of an nsp12 monomer in complex with one nsp7-nsp8 pair and an nsp8 monomer, as was previously observed for SARS-CoV (*9*). In addition to the nsp12-nsp7-nsp8 complex, we also observed single-particle classes corresponding to the nsp12-nsp8 dimer, as well as individual nsp12 monomers, but these do not produce atomic-resolution reconstructions (fig. S2). However, the nsp12-nsp7-nsp8 complex reconstruction provides the structural information for complete structural analysis.

**Fig. 1:**
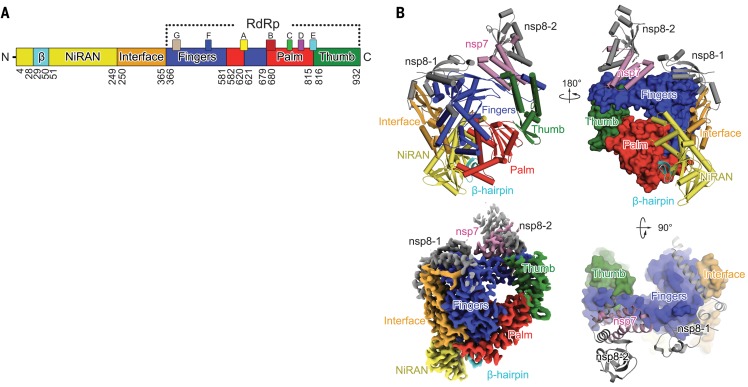
Structure of COVID-19 virus nsp12-nsp7-nsp8 complex. (**A**) Domain organization of COVID-19 virus nsp12. The interdomain borders are labeled with residue numbers. The N-terminal portion with no cryo-EM map density and the C-terminal residues that cannot be observed in the map are not included in the assignment. The polymerase motifs are colored as follows: motif A, yellow; motif B, red; motif C, green; motif D, violet; motif E, cyan; motif F, blue; and motif G, light brown. (**B**) Ribbon diagram of COVID-19 virus nsp12 polypeptide chain in three perpendicular views. Domains are colored the same as in (A). The individual nsp8 (nsp8-1) bound to nsp12 and that in the nsp7-nsp8 pair (nsp8-2) are shown in gray; the nsp7 is in pink. The bottom left panel shows an overview of the cryo-EM reconstruction of the nsp12-nsp7-nsp8 complex.

**Fig. 2:**
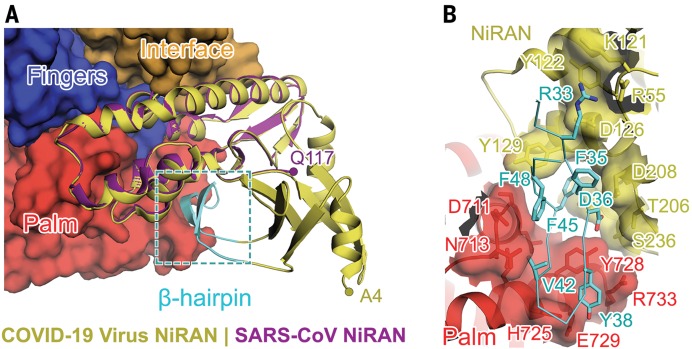
Structure of N-terminal NiRAN domain and β hairpin. (**A**) Overall structure of the N-terminal NiRAN domain and β hairpin of COVID-19 virus nsp12. The N-terminal NiRAN domain and β hairpin of COVID-19 virus nsp12 are shown as yellow and cyan cartoons, respectively, whereas the other regions of COVID-19 virus nsp12 are shown as a molecular surface with the same color scheme used in Fig. 1. The NiRAN domain of SARS-CoV nsp12 is superimposed to its counterpart in COVID-19 virus nsp12 and is shown in purple. (**B**) Key interactions between the β hairpin and other domains. The β hairpin is shown as a cyan tube with its key residues in stick mode. These have the closest contacts with other domains of COVID-19 virus nsp12. The interacting residues in the palm and fingers subdomain of the RdRp domain and the NiRAN domain are identified by the labels. Single-letter abbreviations for the amino acid residues are as follows: A, Ala; C, Cys; D, Asp; E, Glu; F, Phe; G, Gly; H, His; I, Ile; K, Lys; L, Leu; M, Met; N, Asn; P, Pro; Q, Gln; R, Arg; S, Ser; T, Thr; V, Val; W, Trp; and Y, Tyr.

The structure of the COVID-19 virus nsp12 contains a right-hand RdRp domain (residues S367 to F920) and a nidovirus-specific N-terminal extension domain (residues D60 to R249) that adopts a nidovirus RdRp-associated nucleotidyltransferase (NiRAN) (*10*) architecture. The polymerase domain and NiRAN domain are connected by an interface domain (residues A250 to R365) (Fig. 1, A and B). An additional N-terminal β hairpin (residues D29 to K50), built with the guidance of an unambiguous cryo-EM map (fig. S3A), inserts into the groove clamped by the NiRAN domain and the palm subdomain in the RdRp domain (Fig. 2). The nsp7-nsp8 pair shows a conserved structure similar to that of the SARS-CoV nsp7-nsp8 pair (*9*, *11*). The orientation of the N-terminal helix of the separate nsp8 monomer bound to nsp12 is shifted compared with that in the nsp7-nsp8 pair (fig. S4A). The 13 additional amino acid residues resolved at the N-terminal of nsp8 show that the long shaft of its well-known golf club shape is bent (fig. S4B).

The overall architecture of the COVID-19 virus nsp12-nsp7-nsp8 complex is similar to that of SARS-CoV with a root mean square deviation (RMSD) value of 0.82 for 1078 Cɑ atoms (fig. S4C). However, there are key features that distinguish the two. The cryo-EM map allowed us to build the complete structure of COVID-19 virus nsp12, including all residues except S1 to D3 and G897 to D910. In contrast, the first 116 residues were not resolved in SARS-CoV nsp12 (*9*). The portion of the NiRAN domain resolved in SARS-CoV (residues 117 to 249) is composed of six helices with a three-stranded β sheet at the N terminus (*9*) (Fig. 2A). In the COVID-19 virus structure, we additionally resolved residues A4 to R118. These constitute a structural block with five antiparallel β strands and two helices. Residues N215 to D218 form a β strand in COVID-19 virus nsp12, whereas these residues are less ordered in SARS-CoV nsp12. This region makes contact with the strand that includes residues V96 to A100, thus contributing to the stabilization of its conformation. As a result, these four strands form a compact semi–β barrel architecture. Therefore, we identify residues A4 to T28 and Y69 to R249 as the complete coronaviral NiRAN domain. With the resolution of N-terminal residues, we are also able to identify an N-terminal β hairpin (D29 to K50; Figs. 1A and 2A). This β hairpin inserts into the groove clamped by the NiRAN domain and the palm subdomain in the RdRp domain and forms a set of close contacts to stabilize the overall structure (Fig. 2B and fig. S5). We have also observed C301 to C306 and C487 to C645 form disulfide bonds in the absence of DTT (dataset 1). However, in the presence of DTT (dataset 2), chelated zinc ions are present in the same location as that observed in SARS-CoV (fig. S3B).

The polymerase domain adopts the conserved architecture of the viral polymerase family (*12*) and is composed of three subdomains: a fingers subdomain (residues L366 to A581 and K621 to G679), a palm subdomain (residues T582 to P620 and T680 to Q815), and a thumb subdomain (residues H816 to E920) (Fig. 1). The catalytic metal ions, which are observed in several structures of viral polymerases that synthesize RNA (*13*, *14*), are not observed in this work in the absence of primer-template RNA and nucleoside triphosphates (NTPs).

**Fig. 3:**
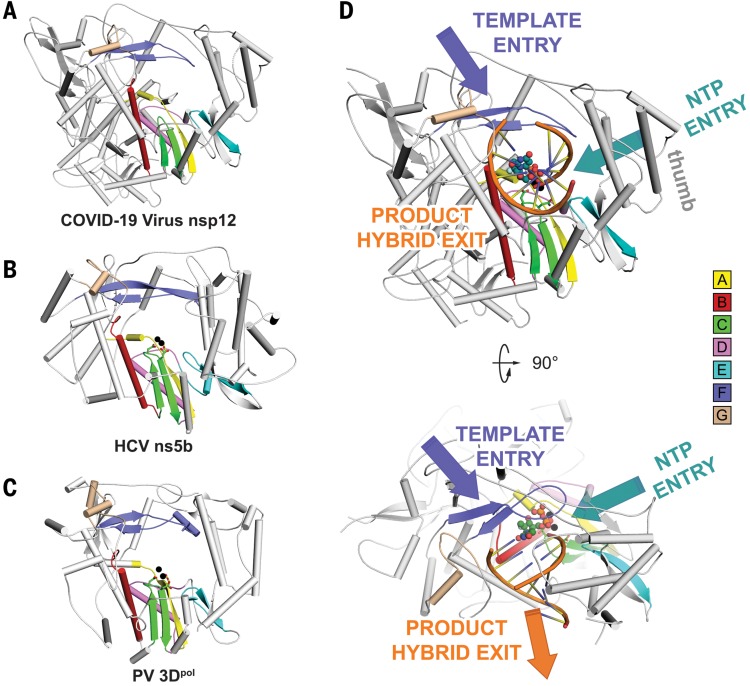
The RdRp core region. (**A** to **C**) Structural comparison of COVID-19 virus nsp12 (A), HCV ns5b (PDB ID: 4WTG) (*13*) (B), and PV 3D^pol^ (PDB ID: 3OLB) (*14*) (C). The three structures are displayed in the same orientation. The polymerase motifs (motifs A to G) have the same color scheme used in Fig. 1A. (**D**) The template entry, NTP entry, and product hybrid exit paths in COVID-19 virus nsp12 are labeled in slate, deep teal, and orange colors, respectively. Two catalytic manganese ions (black spheres), pp-sofosbuvir (dark green spheres for carbon atoms), and primer template (orange) from the structure of HCV ns5b in complex pp-sofosbuvir (PDB ID: 4WTG) (*13*) are superposed to COVID-19 virus nsp12 to indicate the catalytic site and nucleotide binding position.

The active site of the COVID-19 virus RdRp domain is formed by the conserved polymerase motifs A to G in the palm domain and configured like other RNA polymerases (Figs. 1A and 3A and fig. S6). Motif A, composed of residues 611 to 626 (TPHLMGWDYPKCDRAM), contains the classic divalent-cation–binding residue D618, which is conserved in most viral polymerases including hepatitis C virus (HCV) ns5b (residue D220) and poliovirus (PV) 3D^pol^ (residue D233) (*13*, *14*) (Fig. 3, B and C). Motif C [residues 753 to 767 (FSMMILSDDAVVCFN)] contains the catalytic residues [759 to 761 (SDD)] in the turn between two β strands. These catalytic residues are also conserved in most viral RdRps, e.g., 317 to 319 (GDD) in HCV ns5b and 327 to 329 (GDD) PV 3D^pol^, with the first residue being either serine or glycine.

In this structure, as in other RNA polymerases, the primer-template entry, NTP entry, and nascent strand exit paths are positively charged and solvent accessible, and they converge in a central cavity where the RdRp motifs mediate template-directed RNA synthesis (Fig. 3D). The configurations of the template-primer entry paths, the NTP entry channel, and the nascent strand exit path are similar to those described for SARS-CoV and for other RNA polymerases, such as HCV and PV polymerase (*14*) (Fig. 3, B and C). The NTP entry channel is formed by a set of hydrophilic residues, including K545, R553, and R555 in motif F. The RNA template is expected to enter the active site composed of motifs A and C through a groove clamped by motifs F and G. Motif E and the thumb subdomain support the primer strand. The product-template hybrid exits the active site through the RNA exit tunnel at the front side of the polymerase.

**Fig. 4:**
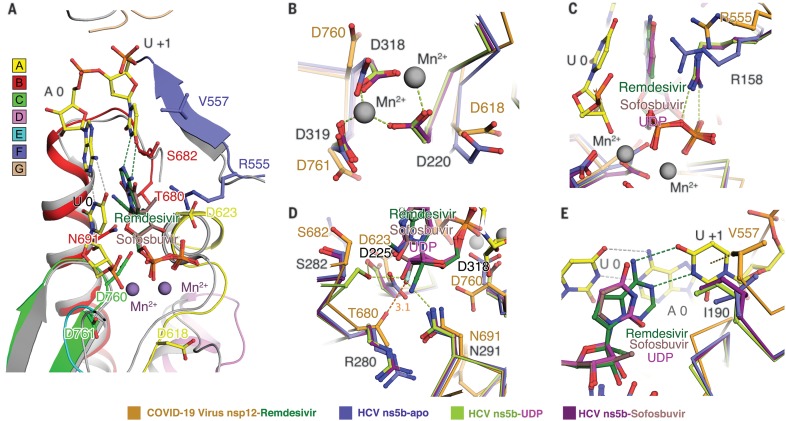
Incorporation model of remdesivir in COVID-19 virus nsp12. (**A**) The polymerase motifs are colored as in Fig. 3. Superposition of the structure of HCV ns5b in complex with pp-sofosbuvir (PDB ID: 4WTG) (*13*) with COVID-19 virus nsp12 shows the possible positions of the two catalytic ions (purple spheres), the priming nucleotide (U 0), template strand, and the incoming pp-remdesivir in nsp12. (**B** to **E**) Structure comparison of HCV apo ns5b or its complex with UDP and pp-sofosbuvir with the COVID-19 virus nsp12.

Remdesivir, the single Sp isomer of the 2-ethylbutyl L-alaninate phosphoramidate prodrug (*15*) (fig. S7), has been reported to inhibit COVID-19 virus proliferation and therefore to have clinical potential (*7*, *8*). We will briefly discuss its possible binding and inhibition mechanism on the basis of the results of this study. The efficacy of chain-terminating nucleotide analogs requires viral RdRps to recognize and successfully incorporate the active form of the inhibitors into the growing RNA strand. Sofosbuvir (2′-F-2′-C-methyluridine monophosphate) is a prodrug that targets HCV ns5b and has been approved for the treatment of chronic HCV infection (*16*). It acts by binding to the catalytic site of HCV ns5b polymerase (*12*, *16*). Given that remdesivir and sofosbuvir are both nucleotide analogs and given the structural conservation of the catalytic site between COVID-19 virus nsp12 and HCV ns5b polymerase (*13*, *16*) (fig. S7), we modeled remdesivir diphosphate binding to COVID-19 virus nsp12 on the basis of superposition with sofosbuvir bound to HCV ns5b (Fig. 4A and fig. S4D). Overall, we found that the nsp12 of COVID-19 virus has the highest similarity with the apo state of ns5b. Given the conformational changes of ns5b in apo, elongation, and inhibited states, it appears that catalytic residues D760, D761, and the classic D618 will undergo a conformational change to coordinate the divalent cations (Fig. 4B). The latter will anchor the phosphate group of the incoming nucleotide or inhibitors together with the allosteric R555 in motif F (Fig. 4C). In the structures of the HCV ns5b elongation complex or its complex with diphosphate sofosbuvir (pp-sofosbuvir), a key feature is that the incorporated pp-sofosbuvir interacts with N291 (equivalent to N691 in COVID-19 virus). However, because of a fluorine substitution on its sugar moiety, pp-sofosbuvir is not capable of joining the hydrogen bonding network with S282 and D225 (Fig. 4D), which is necessary to stabilize the incoming natural nucleotide (*13*). However, remdesivir keeps an intact ribose group, so it may be able to use this hydrogen bond network like a native substrate. Additionally, T680 in COVID-19 virus nsp12 is also likely to form hydrogen bonds with the 2′ hydroxyl of remdesivir and, of course, with incoming natural NTP (Fig. 4D). Moreover, the hydrophobic side chain of V557 in motif F is likely to stack with and stabilize the +1 template RNA uridine base to base pair with the incoming triphosphate remdesivir (ppp-remdesivir) (Fig. 4E).

The rapid global spread of COVID-19 virus has emphasized the need for the development of new coronavirus vaccines and therapeutics. The viral polymerase nsp12 appears to be an excellent target for new therapeutics, especially given the fact that lead inhibitors already exist in the form of compounds such as remdesivir. Considering the structural similarity of nucleoside analogs, the binding mode and inhibition mechanism discussed here may also be applicable to other similar drugs or drug candidates such as favipiravir, which has proven effective in clinical trials (*17*). This target, in addition to other promising drug targets such as the main protease, could support the development of a cocktail of anti-coronavirus treatments that potentially can be used for the discovery of broad-spectrum antivirals.

## Supplementary Materials

science.sciencemag.org/content/368/6492/779/suppl/DC1 Materials and Methods Figs. S1 to S7 Table S1 References (*18*–*24*) Movie S1

## Appendix

View/request a protocol for this paper from *Bio-protocol*.
